# Overweight in family members of probands with ADHD

**DOI:** 10.1007/s00787-019-01331-7

**Published:** 2019-04-19

**Authors:** Pauline M. Geuijen, Jan K. Buitelaar, Ellen A. Fliers, Athanasios Maras, Lizanne J. S. Schweren, Jaap Oosterlaan, Pieter J. Hoekstra, Barbara Franke, Catharina A. Hartman, Nanda N. Rommelse

**Affiliations:** 1 Karakter Child and Adolescent Psychiatry University Center, Reinier Postlaan 12, 6525 GC Nijmegen, The Netherlands; 2 Department of Psychiatry, Donders Institute for Brain, Cognition and Behaviour, Centre for Neuroscience, Radboud University Medical Center, Nijmegen, The Netherlands; 3 Department of Cognitive Neuroscience, Donders Institute for Brain, Cognition and Behaviour, Centre for Neuroscience, Radboud University Medical Centre, Nijmegen, The Netherlands; 4 Virenze Child and Adolescent Psychiatry, Gorinchem, The Netherlands; 5 Yulius Academy, Yulius Mental Health Organization, Barendrecht, The Netherlands; 6 Department of Psychiatry, University Medical Center Groningen, University of Groningen, Groningen, The Netherlands; 7 Clinical Neuropsychology Section, Vrije Universiteit Amsterdam, Amsterdam, The Netherlands; 8 Emma Children’s Hospital, Academic Medical Center, Amsterdam, The Netherlands; 9 Department of Pediatrics, Vrije Universiteit Medical Center, Amsterdam, The Netherlands; 10 Accare University Child and Adolescent Psychiatry Center, Groningen, The Netherlands; 11 Department of Human Genetics, Donders Institute for Brain, Cognition and Behaviour, Centre for Neuroscience, Radboud University Medical Center, Nijmegen, The Netherlands

**Keywords:** Attention-deficit/hyperactivity disorder, Overweight, Family, Child, Adolescent

## Abstract

The widely reported association between ADHD and overweight may be attributable to genetic and environmental factors also present in unaffected family members. Therefore, the purpose of this study was to examine the association between ADHD and overweight within families. A cohort was used of families with at least one member with ADHD, recruited as part of the Dutch node of the International Multicenter ADHD Genetics (IMAGE) study, with assessments taking place between 2003 and 2006, 2009 and 2012, and 2013 and 2015. The three assessment waves yielded *N* = 1828 youth assessments and *N* = 998 parent assessments from *N* = 447 unique families. Overweight was defined as a body mass index (BMI) ≥ 85th percentile for youth of the same age and sex; overweight in adults as a BMI ≥ 25. Effects of age, gender, and medication use (psychostimulants, antipsychotics, and melatonin) were taken into account. Generalized estimation equations were used to correct for within-family and within-subject correlations. There was no difference in risk between ADHD-affected youth and their unaffected siblings (OR 0.92, 95% CI 0.78–1.09). However, compared to population prevalence data, all ADHD family members alike were at increased risk for being overweight: ADHD-affected youth (OR 1.33, 95% CI 1.13–1.59), unaffected siblings (OR 1.73, 95% CI 1.45–2.08), mothers (OR 1.74, 95% CI 1.40–2.17) and fathers (OR 1.78, 95% CI 1.46–2.15). Parental overweight—but not parental ADHD—was predictive of offspring overweight (mothers OR 1.40; 95% CI 1.14–1.73, fathers OR 1.83; 95% CI 1.41–2.36). Being overweight runs in ADHD families, yet is not specifically linked to ADHD within families. Shared unhealthy lifestyle factors (including nutrition, sleep, exercise, stress) as well as genetic factors shared by family members likely explain the findings.

**Electronic supplementary material:**

The online version of this article (10.1007/s00787-019-01331-7) contains supplementary material, which is available to authorized users.

## Introduction

Is there a relationship between attention-deficit/hyperactivity disorder (ADHD) and being overweight? This has been the central question for many small- and large-scale studies during the last 2 decades. Two recent meta-analyses concluded that people with ADHD have a 20–30% increased chance of being overweight compared to population prevalence rates [1, 2]. Inconsistent conclusions were drawn regarding gender effects [1, 2]. Vice versa, it has also been shown that overweight individuals—compared to individuals with a healthy weight—are more likely to show ADHD symptoms [3–5].

Various mechanisms have been proposed to explain the association between ADHD and overweight. ADHD may causally lead to overweight through impulsive (binge) eating [6] since ADHD is often characterized by emotional lability, impulsivity, and disturbances in sleep and circadian rhythm [7, 8]. There is no direct evidence for this hypothesis, but there is a substantial amount of indirect evidence. The prevalence of binge eating symptoms and bulimia nervosa is higher in patients with ADHD [9]. Similarly, patients with bulimia nervosa have an increased chance of having (a childhood history of) ADHD and executive control problems [10–12]. Alternatively, instead of a causal association between ADHD and overweight, shared etiologies between ADHD and overweight may be key to understanding the association between both. Currently, to the best of our knowledge, one twin and sibling study has directly tested the contribution of shared genetic and environmental factors for both phenotypes in young and older adulthood [13]. They found a significant role for unique environmental influences explaining the association between both phenotypes in males and genetic influences contributing to the association between both phenotypes in females. Similarly, another study also suggested that the association between ADHD and binge eating in females was largely explained by shared genetic risk factors [14], suggesting the mechanisms underlying the association between ADHD and overweight may be different in males and females. Several molecular genetic studies have reported common candidate genes for ADHD and increased BMI [15–17]. In addition, a large-scale genetic correlation between both phenotypes was reported based on genome-wide association studies of common genetic variants, but potential sex moderating effects were not reported [18]. Apart from a shared genetic background, both phenotypes also show very similar relationships with adverse environmental factors such as low socio-economic status, parental psychopathology, and poor nutritional and exercise habits [19–23]. A recent report even argued that the link between ADHD symptoms and increased body weight could be completely explained by cumulative psychosocial risks, and highlighted the importance of chronic stress as a trigger for both conditions [19]. In a recent comprehensive review on potential mechanism explaining the association between ADHD and overweight [24], including genetic factors, fetal programming, executive dysfunctions, psychosocial stress, factors directly related to energy balance, and sleep patterns alterations, it was concluded that physical activity and eating patterns as possible and most direct causes of weight gain do not seem to fully explain the link and need to be integrated in a broader biopsychosocial model. It was proposed that ADHD and obesity might have a common neurocognitive phenotype, characterized by deficits in hot executive functions, associated with impulsivity and difficulties in postponing gratification [24]. Fetal programming is proposed to be an important element in this model, with environmental agents such as maternal stress, maternal smoking, and drinking alcohol during pregnancy disrupting the development of executive functions.

To test this model, this study employs a within-family design to examine mechanisms underlying the association between ADHD and being overweight in ADHD-affected and -unaffected family members taking into account age, gender, and medication use since these have been reported as moderating factors [25–28]. A family-based study design including unaffected family members of patients with ADHD provides unique opportunities. It allows to investigate whether overweight in ADHD is associated with the presence of ADHD or with shared familial factors. If the association between ADHD and overweight is indeed primarily linked to deficits in hot executive functions, characterized by impulsivity and difficulties in postponing gratification, it is expected that overweight will be primarily present in family members meeting criteria for ADHD. However, if the association between ADHD and overweight is primarily explained by factors shared by family members with and without ADHD, a similar increased prevalence of being overweight in family members affected and unaffected by ADHD is expected compared to population prevalence rates. In addition, the predictive effect of parental BMI on child BMI is expected to be larger than the predictive effect of parental ADHD on child BMI. Remarkably and to the best of our knowledge, only one prior study addressed this issue in a family design, albeit indirectly [29]. In a large population-based sample (*N* = 11,159), the association between children’s (6–17 years) ADHD symptoms and body mass index (BMI) status was strongly attenuated when parental BMI was accounted for [29]. This may indicate that the association between ADHD symptoms and overweight/obesity is due to confounding by family background. However, parental ADHD symptoms were not taken into account, preventing a direct comparison of the effects of parental BMI and parental ADHD status on offspring weight status. One previous study included unaffected siblings for studying the relationship between overweight and ADHD within families [5]. However, parental overweight and ADHD symptoms were not included in this study.

## Methods

### Participants

Families (*N* = 369) were recruited as part of the Dutch node of the International Multicenter ADHD Genetics (IMAGE) study, with assessments taking place between 2003 and 2006 (wave 1), see Figure S1. All families were invited for a second assessment (wave 2), which took place between 2009 and 2012. Follow-up rate was 75.9% (*N* = 280 families). New families (*N* = 45) were recruited at the second assessment wave as well [30]. Recently, a proportion of the families (*N* = 184) was invited for the third assessment (wave 3; 2013–2015) with a follow-up rate of 47.3% (*N* = 87). Again, new families (*N* = 33) were added to the sample. The three assessment waves yielded *N* = 1828 youth assessments and *N* = 998 parent assessments from *N* = 447 unique families, for a description of the full sample see Table 1. A family-based control cohort was available with children (not parents) measured during the same assessment waves comprising a total of *N* = 361 assessments from *N* = 136 unique families.

**Table 1:** Sample description of all assessments (*N* = 1828) for all youth individuals (*N* = 998; 31.7% was measured once, 53.5% was measured twice, 14.8% was measured three times) within the NeuroIMAGE cohort

	ADHD-affected youth assessments		Unaffected youth assessments		*t* test
	*N*	*M* (SD)/ %	*N*	*M* (SD)/ %	*p* value
Age in years	1037	14.3 (4.3)	791	15.3 (5.2)	< 0.0001
Male gender	1037	72	791	45	< 0.0001*
IQ	997	99 (17)	752	104 (16)	< 0.0001
CPRS *T* scores					
Inattentive	1009	68 (11)	751	50 (9)	< 0.0001
Hyperactive	1010	73 (14)	752	51 (11)	< 0.0001
Total	1010	73 (12)	752	50 (10)	< 0.0001
CORS *T* scores					
Inattentive	978	65 (11)	742	50 (10)	< 0.0001
Hyperactive	976	65 (14)	742	49 (11)	< 0.0001
Total	977	67 (12)	742	49 (10)	< 0.0001
Parental age in years					
Mother	925^a^	43.2 (5.3)	665^a^	43.9 (5.6)	0.0060
Father	930^a^	45.8 (6.0)	668^a^	46.0 (6.1)	0.25
Parental education in years					
Mother	784^a^	11.4 (2.3)	593^a^	11.3 (2.0)	0.20
Father	660^a^	11.6 (2.8)	499^a^	11.7 (2.8)	0.27
Parental ADHD symptom presence					
Mother	922^a^	1.3	661^a^	0.9	0.45*
Father	858^a^	1.0	615^a^	0.5	0.28*
Parental BMI in kg/m^2^					
Mother	726^a^	27.3 (5.3)	543^a^	27.6 (5.3)	0.15
Father	647^a^	27.7 (4.4)	488^a^	27.6 (4.5)	0.35

Parental data are presented separately for assessments of ADHD-affected and -unaffected youth, but since at least two children/adolescents per family participated, parental data by definition largely overlaps

*CPRS* Conners’ Rating Scale completed by Parents, *CORS* Conners’ Rating Scale completed by Others (teacher or self-report), *N* number, *M* mean, *SD* standard deviation

^a^Parental data were collected for waves 1 and 2 of the NeuroIMAGE cohort (IMAGE and NeuroIMAGE)

*χ^*2*^ test was used

Conner’s long-version parent and teacher questionnaires (youth < 18 years) or parent and self-report (youth ≥ 18 years) were used to screen all youth [31–33]. *T* scores ≥ 63 on the DSM-IV ADHD subscale inattention (L), hyperactivity/impulsivity (M), and total symptoms (N) were considered clinical. Youth scoring clinically on any of these subscales were administered a semi-structured, standardized, investigator-based interview: the parental account of children’s symptoms (PACS) (wave 1) or the schedule for affective disorders and schizophrenia for school-age children—present and lifetime version (K-SADS [34]; waves 2 and 3, respectively). ADHD types/presentations (combined, predominantly inattentive, or predominantly hyperactive/impulsive) were established according to DSM-IV-TR criteria (first assessment) or DSM-5 criteria (second and third assessment) (for full description of diagnostic procedures see [30]).

Self-reported ADHD symptoms in the parents were measured with either the ADHD rating scale (wave 1) [35] or the Conner’s self-report (waves 2 and 3) [32]. Ratings were dichotomized into presence (above cut-off) or absence (below cut-off) for ADHD.

### Measures

Body weight and height were measured by professionals in the hospital. The individuals were clothed but wore no shoes or coats. Weight was measured to 0.1 kg using an electronic flat scale (Seca 877, Seca GmbH & Co. KG, Hamburg, Germany), height was determined to the nearest 5 mm using a wall-mounted tape measure (Seca 20, Seca GmbH & Co. KG, Hamburg, Germany). BMI was calculated as weight in kilograms divided by squared height in meters [36]. The classification of youth and their parents as being overweight (including obesity) was based on sex- and age-specific cut-off values of the World Obesity Federation (formerly known as International Obesity Task Force (IOTF)) [37, 38]. Briefly, overweight in youth was defined as a BMI at or above the 85th percentile for youth of the same age and sex. In individuals older than 18 years a BMI of 25 or higher defined being overweight. At the third assessment, parental BMI data were not collected. Population prevalence rates of being overweight in youth were obtained from the Fifth National Growth Study, a sample of 12,104 individuals aged 2–21 years measured during 2009–2010 by community health services [39]. Adult population prevalence rates of being overweight were obtained from the Dutch Health Survey 2014, a sample of 3715 adults aged 31–60 years measured by Statistics Netherlands (CBS) [40].

Full-scale IQ of all youth was estimated by four subtests (vocabulary, similarities, block design, and picture completion) of the WISC/WAIS-III (Wechsler Intelligence Scale for Children or Wechsler Adult Intelligence Scale-III) [41].

For ADHD-affected offspring, we recorded the use of psychostimulants (methylphenidate and atomoxetine), antipsychotics (risperidone), and melatonin up to 4 months prior to the assessment day. Preferably, pharmacy transcripts were used to obtain data on medication use (yes/no). If pharmacy records were not available, self-report questionnaires on current and past medication use were used as binary measures.

Socio-economic status was operationalized by the highest successfully completed education of each parent. This scale contained nine levels, ranging from 0 (no formal education) to 9 (university education) [42, 43].

### Statistical analyses

Analyses were performed using the Statistical Package for Social Sciences, version 22.0 for Windows (IBM Corporation, New York, USA). Generalized estimating equations (GEE) were used to correct for within-family and within-subject correlations (correlated measurements over time). In the first step (default youth model), we predicted youth overweight status (yes/no) by ADHD diagnosis (yes/no), gender (male/female), age (continuous), age^2^ (continuous), and IQ (continuous). In the second step (extended youth model), two-level interactions among predictors were added to the default model predicting youth overweight status (yes/no); predictors were dropped in case of non-significance. In a third step (parental youth model), the following variables were added to the extended model predicting youth overweight status (yes/no): age parent (continuous), education parent (continuous), ADHD score parent above cut-off (yes/no), and overweight status parent (yes/no). Parental data were only available for a subsample of 539 individuals (*N* = 828 assessments). In the last step (affected youth medication model), for ADHD-affected youth, the extended model predicting youth overweight status (yes/no) was extended by adding the following predictors: use of psychostimulants (yes/no), antipsychotics (yes/no), and melatonin (yes/no). Two-level interactions among predictors were added to the medication model and dropped in case of non-significance.

Parental overweight was predicted using ADHD above cut-off (yes/no), gender (male/female), age (continuous), and age^2^ (continuous) as predictors (default parent model). Two-level interactions among predictors were added to the default model and dropped in case of non-significance (extended parent model).

Continuous variables were centered to the median, enabling predictive values to reflect the median population (median youth age = 14.5 years, median estimated IQ = 100, median years of parental education = 10.5 years, median mother’s age = 43.4 years, median father’s age = 45.6 years). All final probability models were created by backward elimination with a significance level of 0.1. Only for two essential youth variables (IQ and BMI), multiple imputation (20 imputations) was used for missing values (< 15%).

Estimates of the extended youth and parent probability models were used to plot overweight probabilities stratified by gender (male/female), family member (mother, father, ADHD-affected child, unaffected sibling), and age (continuous). Youth and adult population prevalence were included in these plots for reference. For youth, these plots ranged till the age of 21 years since population prevalence data were available for youth till the age of 21 years. Odds ratios were calculated to compare the prevalence of overweight in ADHD-affected family members by the overweight population prevalence. Comparisons to the control family cohort are presented in the supplementary material. Correction for multiple testing was applied using the false discovery rate (FDR) [44].

## Results

Demographics of the sample are shown in Table 1. Compared to the unaffected siblings’ assessments, ADHD-affected youth were younger than their unaffected siblings, had lower IQ scores, were more likely male and had—as expected—higher ADHD symptom scores. Parental variables, except for maternal age, were similar for both groups of youth (Table 1). Point prevalence rates of overweight uncorrected for age and sex were 17.6% (ADHD-affected youth), 22.0% (unaffected siblings), 60.4% (mothers) and 72.3% (fathers).

In relation to the main objective whether or not ADHD is associated with overweight within families, the first step (default youth model) showed no main effect of ADHD status on overweight status (OR 0.92, 95% CI 0.78–1.09), i.e., youth with and without ADHD were equally likely to be overweight. In the second step (extended youth model), we found a small ADHD status by age interaction effect such that the risk for being overweight increased with age in youth with ADHD but not in unaffected youth (OR 1.04, 95% CI 1.01–1.07); however, this effect did not survive correction for multiple testing. No other significant or near-significant two-level interactions among predictors were found in the extended model.

In the third step (parental youth model), adding parental variables to the model predicting youth overweight status did not change the predictive value of the default model variables (ADHD diagnosis, gender, age, age^2^, and IQ). Both maternal and paternal overweight predicted overweight in their offspring (mothers OR 1.40; 95% CI 1.14–1.73, fathers OR 1.83; 95% CI 1.41–2.36) (Table 2). Maternal education was predictive of offspring overweight (OR 1.05; 95% CI 1.00–1.10), but this effect did not survive correction for multiple testing. Parental age and ADHD above cut-off were not predictive of being overweight in offspring. In the last step (affected youth medication model), the use of psychostimulants, antipsychotics, or melatonin did not change the predictive value of the default model variables and was not predictive of being overweight in ADHD-affected youth (OR 0.98, 95% CI 0.76–1.26; OR 0.81, 95% CI 0.49–1.34; OR 0.74, 95% CI 0.50–1.07, respectively), nor were interactions between them.

**Table 2:** Probability models for being overweight in youth within the NeuroIMAGE cohort derived from generalized estimating equations

	OR (95% CI)	
**Default youth model**		
Intercept	0.50 (0.43; 0.59)^a^	
ADHD diagnosis	0.93 (0.79; 1.10)	
Male gender	0.73 (0.61; 0.86)^a^	
Age (centered)	1.00 (1.00; 1.03)	
Age (centered)^2^	1.00 (1.00; 1.01)	
IQ (centered)	0.99 (0.98; 1.01)	
*N assessments*		1749
**Extended youth model**		
ADHD diagnosis × male gender	–	
ADHD diagnosis × age (centered)	1.04 (1.01; 1.07)^a^	
Male gender × age (centered)	–	
*N assessments*		1749
**Parental youth model**		
Maternal age (centered)	–	
Paternal age (centered)	–	
Maternal education (centered)	1.05 (1.00; 1.10)^a^	
Paternal education (centered)	–	
Maternal ADHD score above cut-off	–	
Paternal ADHD score above cut-off	–	
Maternal overweight	1.40 (1.14; 1.73)^a^	
Paternal overweight	1.83 (1.41; 2.36)^a^	
*N assessments*		828
**Affected youth medication model**		
Psychostimulant use	0.98 (0.76; 1.26)	
Antipsychotic use	0.81 (0.49; 1.34)	
Melatonin use	0.74 (0.50; 1.07)	
Psychostimulant use × male gender	–	
Psychostimulant use × age (centered)	–	
Antipsychotic use × male gender	–	
Antipsychotic use × age (centered)	–	
Melatonin use × male gender	–	
Melatonin use × age (centered)	–	
*N assessments*		641

^a^Significant after FDR correction

Parental overweight (default parent model) was predicted by gender but not age: fathers were found to be more often overweight than mothers (OR 1.40; 95% CI 1.19–1.66; Table 3). ADHD above cut-off did not predict overweight in parents.

**Table 3:** Probability models for being overweight in parents within the NeuroIMAGE cohort derived from Generalized Estimating Equations

	OR (95% CI)	
**Default parent model**		
Intercept	1.37 (1.21; 1.55)	
ADHD score above cut-off	1.34 (0.52; 3.46)	
Male gender	1.40 (1.19; 1.66)^a^	
Age (centered)	1.01 (1.00; 1.03)	
Age (centered)^2^	1.00 (1.00; 1.00)	
*N assessments*		970
**Extended parent model**		
ADHD score above cut-off × male gender	–	
ADHD score above cut-off × age (centered)	–	
Male gender × age (centered)	–	
*N assessments*		970

^a^Significant after FDR correction

**Fig. 1:**
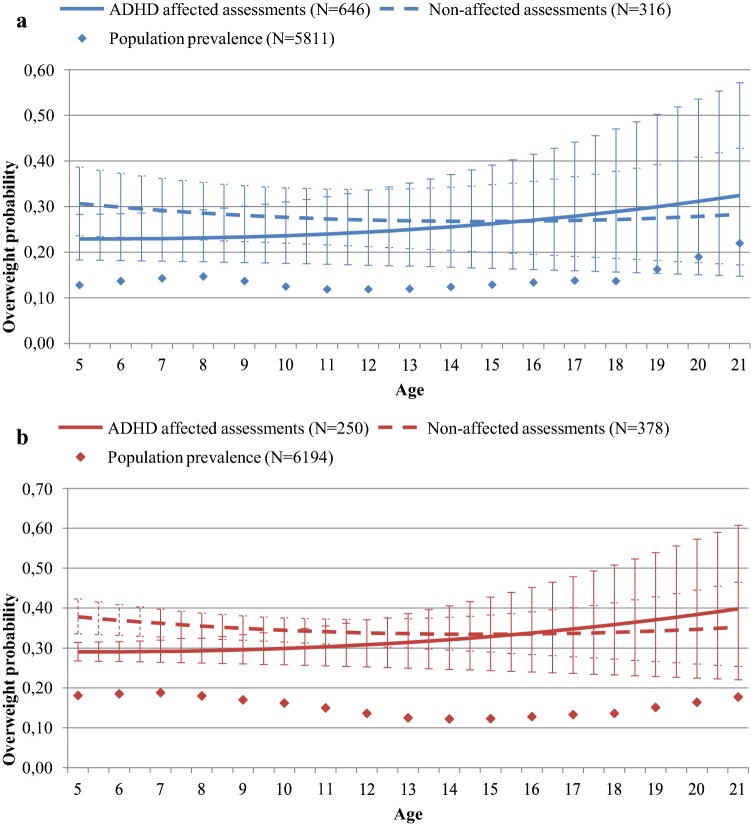
**a** Males and **b** females. Prediction of overweight probability in males and females stratified by ADHD diagnosis (*N* = 962 assessments, derived from 571 males, and *N* = 628 assessments, derived from 374 females, respectively). Error bars represent 95% confidence interval for predicted probabilities. Population prevalence of being overweight was derived from the Fifth National Growth Study (2009–2010). Note: prediction of overweight probabilities in adolescents older than 21 years was not included in this figure since there were no reliable reference data available for this age group

**Fig. 2:**
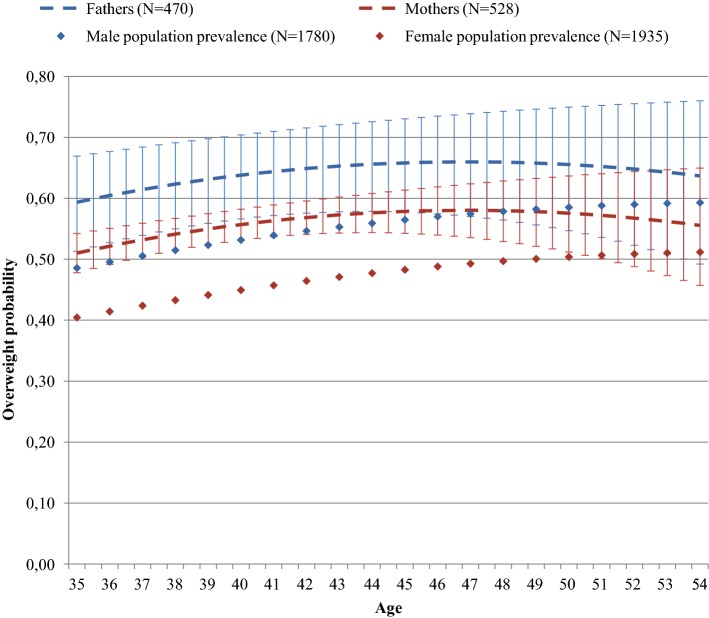
Prediction of overweight probability in parents (*N* = 998 assessments, derived from 655 parents). Error bars represent 95% confidence interval for predicted probabilities. Population prevalence of being overweight was derived from the Dutch Health Survey 2014

As shown in Figs. 1 and 2 (separately shown for males and females), compared to the national population prevalence estimates, a higher prevalence of being overweight was measured in all groups compared to the normative data: ADHD-affected youth (OR 1.33, 95% CI 1.13–1.59); unaffected siblings (OR 1.73, 95% CI 1.45–2.08); fathers (OR 1.74, 95% CI 1.40–2.17); mothers (OR 1.78, 95% CI 1.46–2.15). Findings were similar (albeit less significant) when ADHD-affected youth and unaffected siblings were compared to a consecutively measured control cohort of youth (ADHD: OR 1.12, 95% CI 0.91–1.38; unaffected siblings: OR 1.73, 95% CI 1.27–2.34; Tables S4a, S4b, available online).

## Discussion

In a large family-based cohort, we investigated the extent to which associations between ADHD and being overweight are attributable to shared familial factors or specifically linked to ADHD status. We compared the risk of being overweight in individuals with ADHD, their unaffected siblings, and their parents with population normative data and a control family-based cohort. The three assessment waves yielded *N* = 1828 youth assessments and *N* = 998 parent assessments from *N* = 447 unique families. Results indicated that individuals with ADHD showed overweight at an increased prevalence when compared to the general population and the control cohort, but not when compared to their unaffected siblings. Unaffected siblings, mothers, and fathers of individuals with ADHD were just as likely to be overweight as their family member with ADHD. Parental overweight—but not parental ADHD—was predictive of offspring overweight. Age, gender, use of psychostimulants, antipsychotics, and melatonin did not predict the risk for being overweight in ADHD-affected individuals.

Results of the current study suggest that increased prevalence rates of being overweight in ADHD-affected individuals [1, 2, 45–47] are likely based on factors shared in families. Similar to the results of a large case registry study which showed increased risk for ADHD in families with at least one family member being overweight [5], our study showed an increased risk for overweight in families with at least one family member with ADHD. Thus, there seems to be a common cause in the family environment rather than a causal relationship between ADHD and overweight. This is not in line with a recent review on ADHD and overweight [24], hypothesizing that the association between ADHD and overweight may be primarily linked to deficits in hot executive functions, characterized by impulsivity and difficulties in postponing gratification. The results are in line with another study suggesting the association between ADHD and overweight was completely explained by cumulative psychosocial risks [19]. Prime candidate environmental factors that increase the risk for both ADHD and overweight and that are shared by family members include inadequate health behaviors, foremost poor nutritional habits and lack of physical activity [48–51] as well as chronic stress [19]. Poor nutritional habits, lack of physical activity, and chronic stress tend to cluster with each other and within families [19], particularly in families with disadvantaged backgrounds [52]. Each of these factors is known to have direct (enhanced eating) and indirect detrimental effects on weight, and more broadly on physical and mental health [21, 29, 53], e.g., by impacting inflammatory and immune systems and subcellular metabolism [54].

In addition to inadequate health behaviors, genetic factors shared by family members may also account for the link at a population level between ADHD and overweight. Candidate gene studies [15–17] and large-scale genetic correlation based on genome-wide association studies of common genetic variants [18] have shown that genetic factors influencing, e.g., food responsiveness, cognitive and emotional control, dopamine neurotransmission, and circadian rhythm may increase the risk for ADHD and overweight. These variants are likely more prevalent in families where ADHD and/or overweight are present and may explain why some family members develop ADHD, others overweight and others a combination of both. So common genes may be partly responsible for an obesogenic family environment (e.g., lack of parental structuring and guidance [55]) and this same environment increases the risk for ADHD [56, 57]. A recent study suggest that potential genetic mechanisms underlying the association between ADHD and overweight may be sex specific and more pronounced in females than males [13]. However, the current findings were not moderated by sex and suggest that there is a risk of overestimating the association between ADHD and weight since no association between ADHD and overweight was present when correcting for the combined genetic and environmental factors that are shared between affected and unaffected family members. Future work in twin studies may further tease apart the genetic and shared environmental influences accounting for the association between the two phenotypes. It has further been shown recently that gene × environment interactions are of increasing importance for BMI with increasing age [58]. Taking into account both the genetic background and environmental influences in future studies is crucial for a better understanding of the development of overweight and ADHD within families.

Although previous studies reported a negative association between psychostimulant use and BMI [26, 27], our study did not find support for this association. This may suggest that reducing ADHD symptoms with pharmacological treatment is not an effective treatment for overweight status as has been hypothesized previously [59]. Unexpectedly, the use of antipsychotics (known obesogenic medication [60, 61]) was not associated with an increased risk for being overweight either. A potential explanation is that weight gain is a well-known side effect of this medication [28]; individuals at risk for being overweight might, therefore, be less likely to be prescribed antipsychotic or sleep medication, and/or their nutritional patterns might be more strictly monitored. In this study, medication use covered the 4 months prior to assessment but not the time period before, making it possible that the effect of medication on overweight may have been underestimated. It may also be argued that the effect of medication on BMI is small and not likely to make the difference between overweight versus a healthy weight; our dichotomous outcome measure may have reduced the chance of finding an association.

Strengths of the study include the large, family-based cohort allowing for within-family comparisons of the relationship between ADHD and being overweight. Thorough diagnostic data were available for all youth; height and weight were obtained through reliable direct measurements rather than through self-report. Both population prevalence data and a control group were used for comparisons, yielding similar results. As a potential weakness, fewer parental data than offspring data were available since parents were not assessed at all time points. However, results for the subsample with parent data available were fully consistent with the results of the larger sample, indicating that the subsample with parental data was representative of the full sample. Parental ADHD was recorded using self-report, which is less optimal compared to structured diagnostic assessment. Nevertheless, results for parental ADHD data on overweight (i.e., no relationship) were fully in line with the absence of an association between ADHD and overweight in youth using thorough diagnostic procedures for ADHD. Also, several predictors (diagnosis, medication) as well as the outcome measure (overweight) were dichotomized for the purpose of potentially having a clear-cut message based on clinically meaningful variables. Dichotomous predictors and outcome may reduce the chances of finding more subtle effects due to loss of information, yet the large sample size makes it unlikely that this explains the absence of an association between ADHD and being overweight within families. Thereby, comorbid conditions (like depression) associated with overweight were not included in the analyses. However, this does not alter the absence of an association between ADHD and overweight within families.

In conclusion, our results indicate that being overweight runs in ADHD families, yet is not specifically linked to ADHD within these families. Inadequate health behaviors such as poor nutritional habits, lack of physical activity, or chronic stress as well as genetic factors shared by family members likely explain the findings. Gene × environment interaction studies including inadequate health behaviors and chronic stress on mental and physical health should be an important research priority. Given the profound negative health consequences of being overweight, more attention to intervention strategies targeting inadequate health behaviors in families of children with ADHD is a clinically important issue.

## Electronic supplementary material

Below is the link to the electronic supplementary material.

Supplementary material 1 (EPS 5654 kb)

Supplementary material 2 (DOC 45 kb)
