# Supplementary material for: Overweight in family members of probands with ADHD
Source: Eur Child Adolesc Psychiatry. 2019 Apr 19;28(12):1659–69. doi: 10.1007/s00787-019-01331-7 (PMC6861202; doi:10.1007/s00787-019-01331-7)
Supplement: Supplementary file 2 — Supplementary material 2 (DOC 45 kb) [file 787_2019_1331_MOESM2_ESM.doc]

| **Table S1a.** Probability models for being overweight in ADHD affected youth compared to control youth within the NeuroIMAGE cohort derived from Generalized Estimating Equations. | | |
| --- | --- | --- |
| *Default model affected versus control youth* | *OR (95%-CI)* |  |
| Intercept | 0.36 (0.29; 0.44)* |  |
| ADHD diagnosis | 1.12 (0.91; 1.38) |  |
| Male gender | 0.88 (0.73; 1.06) |  |
| Age (centered) | 1.04 (1.01; 1.06)* |  |
| Age (centered)^2^ | 1.00 (1.00; 1.01) |  |
| *N assessments* |  | 1124 |
|  |  |  |
| *Extended model affected versus control youth* |  |  |
| ADHD diagnosis*male gender | - |  |
| ADHD diagnosis*age (centered) | - |  |
| Male gender*age (centered) | - |  |
| *N assessments* |  | 1124 |
| * = significant after FDR correction |  |  |

| **Table S1b.** Probability models for being overweight in ADHD unaffected youth compared to control youth within the NeuroIMAGE cohort derived from Generalized Estimating Equations. | | |
| --- | --- | --- |
| *Default model unaffected versus control youth* | *OR (95%-CI)* |  |
| Intercept | 0.33 (0.25; 0.43)* |  |
| Unaffected sibling | 1.73 (1.27; 2.34)* |  |
| Male gender | 0.97 (0.70; 1.34) |  |
| Age (centered) | 1.04 (0.99; 1.09) |  |
| Age (centered)^2^ | 1.00 (1.00; 1.01) |  |
| *N assessments* |  | 936 |
|  |  |  |
| *Extended model unaffected versus control youth* |  |  |
| ADHD unaffected*male gender | 0.56 (0.37; 0.84)* |  |
| ADHD unaffected*age (centered) | 0.95 (0.91; 1.00)* |  |
| Male gender*age (centered) | - |  |
| *N assessments* |  | 936 |
| * = significant after FDR correction |  |  |
